# Modeling Dislocation Contrasts Obtained by Accurate-Electron Channeling Contrast Imaging for Characterizing Deformation Mechanisms in Bulk Materials

**DOI:** 10.3390/ma12101587

**Published:** 2019-05-15

**Authors:** Hana KRIAA, Antoine GUITTON, Nabila MALOUFI

**Affiliations:** 1 Université de Lorraine – CNRS – Arts et Métiers ParisTech – LEM3, 7 rue Félix Savart, 57070 Metz, France; hana.kriaa@univ-lorraine.fr (H.K.); antoine.guitton@univ-lorraine.fr (A.G.); 2 Laboratory of Excellence on Design of Alloy Metals for low-mAss Structures (DAMAS) – Université de Lorraine, 57073 Metz, France

**Keywords:** ECCI, dislocation contrast, modeled intensity profiles, invisibility criteria, dynamical theory of electron diffraction

## Abstract

Electron Channeling Contrast Imaging (ECCI) is becoming a powerful tool in materials science for characterizing deformation defects. Dislocations observed by ECCI in scanning electron microscope exhibit several features depending on the crystal orientation relative to the incident beam (white/black line on a dark/bright background). In order to bring new insights concerning these contrasts, we report an original theoretical approach based on the dynamical diffraction theory. Our calculations led, for the first time, to an explicit formulation of the back-scattered intensity as a function of various physical and practical parameters governing the experiment. Intensity profiles are modeled for dislocations parallel to the sample surface for different channeling conditions. All theoretical predictions are consistent with experimental results.

## 1. Introduction

The Electron Channeling Contrast Imaging (ECCI) technique is based on the fact that the Back-Scattered Electrons (BSE) signal is highly dependent on the orientation of the incident beam relative to the lattice planes [1]. Therefore, any slight local distortion of the crystal lattice, produced for instance by a dislocation, leads to a BSE intensity (I_BSE_) modulation, thus generating several contrasts such as a bright line on a dark background [2] or a black line on a bright background [3].

For understanding the origin of the dislocation contrasts obtained by ECCI, the two-beam dynamical diffraction theory was adapted from the Transmission Electron Microscopy (TEM) [4,5]. Briefly, inside the crystal, the electron beams are described by a superposition of Bloch waves. The different inelastic scattering events are divided into two categories: those scattered through angles less than 90° (forming the forward-scattering wave) and those scattered through angles greater than 90° (forming the back-scattered wave) [6]. In the multiple scattering model, electrons can be removed from the forward-scattering wave to the back-scattered one and vice-versa. In order to simulate the I_BSE_ profiles for both perfect and imperfect crystal, Spencer et al. [7] and Wilkinson et al. [6,8,9] used this Bloch wave-based model. They showed that, for the perfect crystal, the simulated profiles exhibit the main experimental features of the channeling pattern: bright band and dark edges. The same approach was also used by Reimer [10,11] for a perfect crystal where the multiple scatterings are treated as incoherent. These different approaches were extended to the case of an imperfect crystal containing a dislocation [6,7,8,9] or a stacking fault [12]. Despite their contribution to the theory of defects electron channeling contrasts [7,8,9,10,11,12], detailed calculations leading to an analytical expression of BSE signal as a function of experimental parameters are still missing. Furthermore, in most cases [7,8], theoretical results were not compared to the experiments. This can be illustrated from the dislocation profiles calculated for the Bragg condition, which exhibit an extra-pic of I_BSE_ not observed experimentally [7,8].

To deepen our understanding of the observed channeling contrast of dislocations, we propose an easier way to model the I_BSE_ as a function of physical parameters either relative to the material or governing the ECCI experiment. Our theoretical results show a good agreement with the experiments for several diffraction conditions.

In a crystal, the electronic wave function is a solution of the time independent Schrödinger’s equation and is given by [11]:

(1) $$\Psi (r)\;=\underset{j}{\sum }\varepsilon^{\left(j\right)}\underset{g}{\sum }C_{g}{}^{\left(j\right)}e^{[2\pi i\;\left(k_{0}{}^{\left(j\right)}+g\right)\cdot r]}e^{{[-2\pi q}^{\left(j\right)}z]}$$

The index j refers to the jth wave, $\varepsilon^{\left(j\right)}$ are the excitation amplitudes of the Bloch wave $\psi^{\left(j\right)}$, $C_{g}{}^{\left(j\right)}$ are the amplitudes of the diffracted waves with a wave vector $k_{g}^{\left(j\right)}{\;=\;k}_{0}^{\left(j\right)}\;+\;g$, where $k_{0}^{\left(j\right)}$ is the wave vector of the jth primary wave and g is the diffraction vector. r is the spatial position vector at which the electron intensity is evaluated. The second factor of Equation (1) contains the absorption parameter $q^{\left(j\right)}$ expressing the exponential attenuation of the wave amplitude with increasing depth z.

In order to determine the different coefficients of the Bloch wave function, presented in Equation (1), Reimer used the two-beam condition, i.e., only one set of lattice planes are in the channeling condition. Hence, the BSE signal of a slice of a thickness (dz), located at a depth (z), is given by [11]:

(2) $$\frac{d\eta }{\mathrm{dz}}{\;=\;N\sigma }_{B}{\;\{\psi \psi }^{*}+\;(1-\underset{j}{\sum }{\left|C_{0}{}^{\left(j\right)}\right|}^{2}e^{{[-4\pi q}^{\left(j\right)}z]})\}$$

N is the atom number per unit of volume, $\sigma_{B}$ is the back-scattering cross-section through angles larger than 90°, and ${\psi \psi }^{*}$ is the probability for the Bloch wave to be backscattered at a depth (z). The last terms (in parentheses) in Equation (2) describe the electrons that are removed from the Bloch wave field by scattering before reaching the slice (dz).

The BSE coefficient $\eta_{O.C.}$ is, then, obtained from the integration of Equation (2) in the total interaction depth from z = 0 to z→∞ (labelled Δη in Reimer’s model). O.C. indicates that only the total BSE intensity due to orientation contrast is calculated, while the contributions, due to atomic number and to the surface inclination, are not considered [11]:

(3) $$\eta_{O.C.}\;=\;\frac{{N\sigma }_{B}}{4\pi }{\;\xi }_{0}^{\prime }(-\frac{{s\;\xi }_{g}\;+\frac{\xi_{0}^{\prime }}{\xi_{g}^{\prime }}}{{1+(s\;\xi }_{g}{)}^{2}-(\frac{\xi_{0}^{\prime }}{\xi_{g}^{\prime }}{)}^{2}}+\frac{{s\;\xi }_{g}}{{1+(s\;\xi }_{g}{)}^{2}{+[(1+(s\;\xi }_{g}{)}^{2})(\frac{\xi_{0}^{\prime }}{\xi_{g}}{)]}^{2}})$$

Equation (3) corresponds to the variation of the BSE intensity for a perfect crystal, i.e., the intensity profile of an isolated pseudo-Kikuchi band [7,11,13], where ${\xi \;\prime }_{0}$ and ${\xi \;}^{\prime }{}_{g}$ are the absorption lengths, ${\;\xi }_{g}$ is the extinction distance, and s the deviation parameter.

## 2. Our Theoretical Approach for BSE Intensity Calculation for an Imperfect Crystal

If we consider a column located at a position x away from a dislocation, at position x = 0 and depth z = z_D_ (where z_D_ is the mean depth of the dislocation), the distortion of the lattice planes near the dislocation does not depend on z but only on x, and it is given by $\frac{\partial R}{\partial z}{)}_{{z=z}_{D}}$ (**R** is the displacement field of the crystalline planes) [14].

Therefore, for calculating the I_BSE_ in the case of an imperfect crystal containing a dislocation parallel to the sample surface, independently of the depth z, we take into account a new deviation parameter written by:

(4) $${s\prime \;=\;s\;+s}_{D}\;\mathrm{where}\;s_{D}\;=\;g\cdot \frac{\partial R}{\partial z}{)}_{{z=z}_{D}}$$

s is the deviation from the exact Bragg position in the perfect crystal, which can be experimentally measured [3]. The scalar product $g\cdot \frac{\partial R}{\partial z}{)}_{{z=z}_{D}}$ represents the supplementary deviation $s_{D}$ due to the variation of the incidence angle between the primary beam and the distorted crystalline planes near the dislocation core. Far from the dislocation, the crystal is considered perfect. The planes are not distorted, and the deviation $s_{D}$ becomes zero. Consequently, to take into account the presence of the defect, we substitute s by s′ in the expression of $\eta_{O.C.}$ for a perfect crystal (in Equation (3), which does not depend on z). We obtain:

(5) $$\eta_{O.C.}=\frac{{N\sigma }_{B}}{4\pi }\xi_{0}^{\prime }(-\frac{(s+s_{D}{\left(x\right)}_{z=z_{D}})\xi_{g}+\frac{\xi_{0}^{\prime }}{\xi_{g}^{\prime }}}{1+{((s+s_{D}{\left(x\right)}_{z=z_{D}})\xi_{g}}^{2}-{\left(\frac{\xi_{0}^{\prime }}{\xi_{g}^{\prime }}\right)}^{2}}+\frac{(s+s_{D}{\left(x\right)}_{z=z_{D}})\xi_{g}}{1+{((s+s_{D}{\left(x\right)}_{z=z_{D}})\xi_{g}}^{2}+{\left[(1+{((s+s_{D}{\left(x\right)}_{z=z_{D}})\xi_{g}}^{2}(\frac{\xi_{0}^{\prime }}{\xi_{g}^{\prime }})\right]}^{2}})$$

This allows us to study the variation of the I_BSE_ as a function of x (distance x away from the dislocation core), where the contrast associated to a dislocation is described by $s_{D}$ (containing all the effect of **R**). 

### 2.1. Screw Dislocation

**Figure 1:**
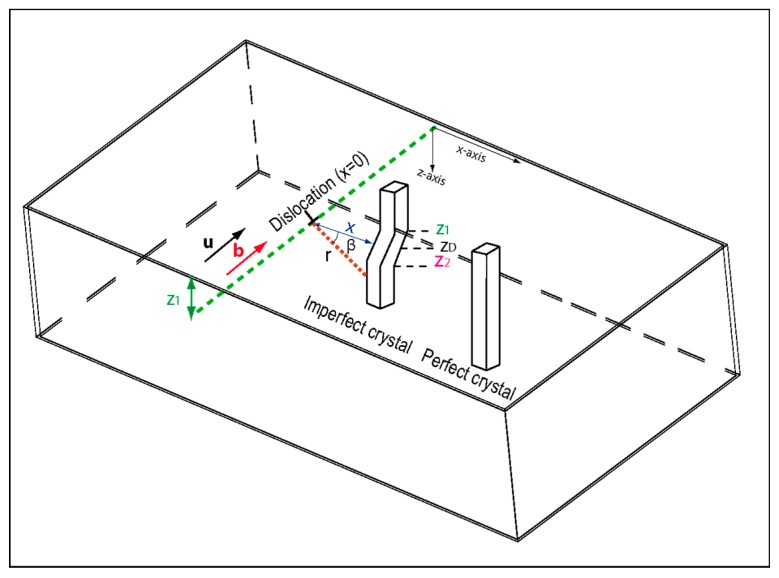
Schematic of a dislocation parallel to the surface and located at a depth z_D_. Deformed planes, perpendicular to the surface, are at a distance x away from the dislocation core.

Figure 1 shows a dislocation parallel to the surface of a bulk sample and located at a depth z_D_. This defect is characterized by a Burgers vector b and a line direction u. At a distance x away from the dislocation core (in x = 0), the crystal plane is deformed. The displacement field $R_{\mathrm{screw}}$ is then defined in polar coordinate (ß) as follows [15]:

(6) $${R\;}_{\mathrm{screw}}\;=\;\frac{b\;ß}{2\pi }\;=\;\frac{b\;}{2\pi }{\;\tan}^{-1}(\frac{{z-z}_{D}}{x})$$

The derivative of ${R\;}_{\mathrm{screw}}$ with respect to the depth z, at a turning point (z = z_D_), is given by:

(7) $${\left(\frac{{dR\;}_{\mathrm{screw}}}{\mathrm{dz}}\right)}_{{z=z}_{D}}\;=\;{\left(\frac{b\;}{2\pi x(1+(\frac{{z-z}_{D}}{x}{)}^{2})}\right)}_{{z=z}_{D}}=\frac{b\;}{2\pi x}$$

Based on this reasoning, the substitution of Equation (7) in Equation (5) allows us to obtain the following expression of $\eta_{O.C.}$:

(8) $$\eta_{O.C.}\;=\;\frac{{N\sigma }_{B}}{4\pi }{\;\xi }_{0}^{\prime }(-\frac{(s+\frac{g\cdot b\;}{2\pi x}{)\xi }_{g}+\frac{\xi_{0}^{\prime }}{\xi_{g}^{\prime }}}{1+((s+\frac{g\cdot b\;}{2\pi x}{)\xi }_{g}{)}^{2}-(\frac{\xi_{0}^{\prime }}{\xi_{g}^{\prime }}{)}^{2}}+\frac{(s+\frac{g\cdot b\;}{2\pi x}{)\xi }_{g}}{1+((s+\frac{g\cdot b\;}{2\pi x}{)\xi }_{g}{)}^{2}+\{[1+((s+\frac{g\cdot b\;}{2\pi x}{)\xi }_{g}{)}^{2}(\frac{\xi_{0}^{\prime }}{\xi_{g}}{)\}}^{2}})$$

Equation (8) gives the variation of the BSE signal as a function of the distance x and the experimental parameters, such as the deviation s and the diffraction vector g.

It should be noted that in this paper, we show profiles modeled in the case of aluminum, where the parameters are: acceleration voltage E = 20 kV, g = (220), the extinction distance $\xi_{g}=\;50\;\mathrm{nm}$, absorption lengths $\xi_{0}^{\prime }=\;140\;\mathrm{nm},$ and $\xi_{g}^{\prime }=\;600\;\mathrm{nm}$ [11]. It should also be mentioned that in all modeled profiles, the background level is taken as reference (at the zero of the ordinate axis). All negative values then correspond to lower BSE intensities than the background level.

#### 2.1.1. Deviation Parameter s = 0

**Figure 2:**
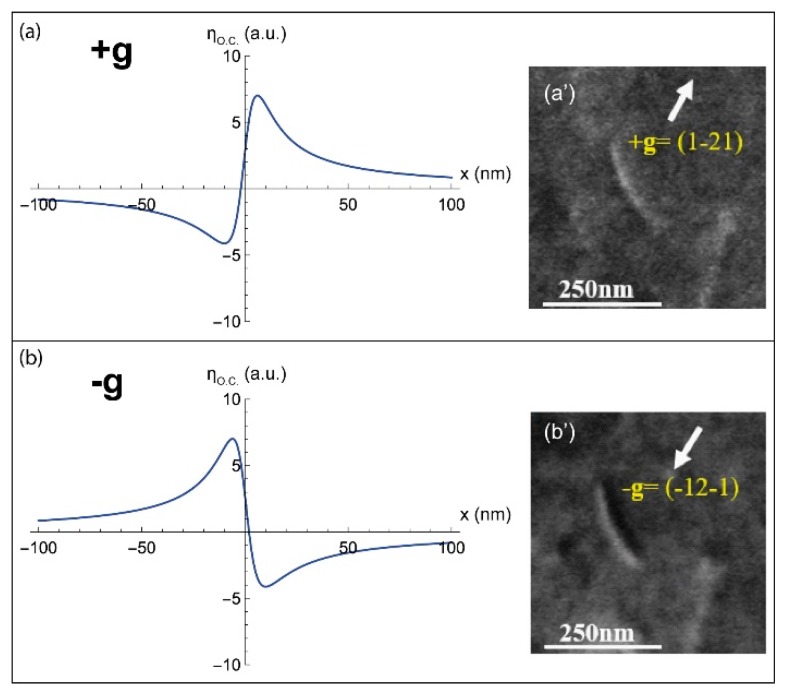
I_BSE_ profiles modeled, for a screw dislocation parallel to the surface, with a deviation parameter s = 0 for the diffractions (**a**) +g and (**b**) −g with their corresponding experimental ECC micrographs (**a’**) and (**b’**).

The theoretical intensity profiles calculated from Equation (8), in the case of a screw dislocation, for the diffraction conditions s = 0 and ±g are represented in Figure 2. Their corresponding experimental ECC micrographs are also shown (Figure 2a’,b’). The **g** and **s** vectors are, respectively, determined experimentally through the pseudo-band indexation of the High Resolution Selected Area Channeling Pattern (HR-SACP) assisted by Electron BackScatter Diffraction (EBSD) experiment [2,3].

For both +g and −g diffractions, the dislocation profiles (Figure 2a,b) are anti-symmetric: a hollow and a peak corresponding to the black and white sides of the dislocation, respectively (Figure 2a’,b’). Moreover, in the case of −g, the extrema are inverted compared to those observed for +g: the peak becomes hollow and vice versa.

Such theoretical results reveal that at Bragg position, a screw dislocation generates a BSE image with black/white sides, which reverse with the inversion of the sign of g. Therefore, the variation of I_BSE_ given by Equation (8) is in good agreement with the experimental observations already reported in literature [3,7].

#### 2.1.2. Deviation Parameter s > 0

**Figure 3:**
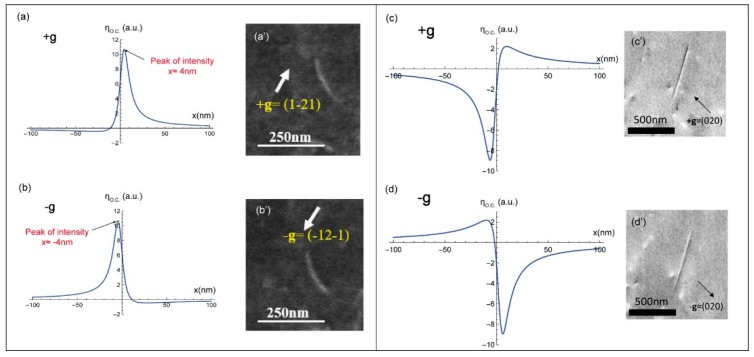
I_BSE_ profiles modeled, for a screw dislocation parallel to the surface for +g and −g, with s > 0 (**a**,**b**), and s < 0 (**c**,**d**), and their corresponding experimental ECC micrographs (**a’**–**d’**).

The I_BSE_ profiles calculated by Equation (8) with a deviation parameter slightly positive ($s\;=\;0.01\;{\mathrm{nm}}^{-1}$) are represented in Figure 3a,b for the +g and −g diffractions, respectively. In this condition (s > 0), both ±g dislocation profiles present one intensity peak only. This is in agreement with the experimental ECC micrographs shown in Figure 3a’,b’: bright line on dark background. Note also that the maximum intensity does not coincide with the exact position of the dislocation core (x = 0 nm) but it is at x ≈ ±4 nm: it is displaced from one side of the dislocation position to the other side when changing from +g to −g. This result is analogous to that obtained in TEM and can be used to characterize a dislocation configuration consisting of two parallel dislocations, such as dipole [3,16].

#### 2.1.3. Deviation Parameter s < 0

The I_BSE_ profiles calculated from our theoretical model for slightly negative deviation parameters ($s\;=\;0.01{\mathrm{nm}}^{-1}$) and ±g diffraction conditions are represented in Figure 3c,d. For the diffraction +g, the curve contains a deep hollow and a peak corresponding to the black and white dislocation sides, respectively (Figure 3c’). This contrast is inverted with the inversion of the sign of g (Figure 3d,d’). For s < 0, the BSE signal emitted from the zone of interest is high: bright background.

### 2.2. Edge Dislocation

**Figure 4:**
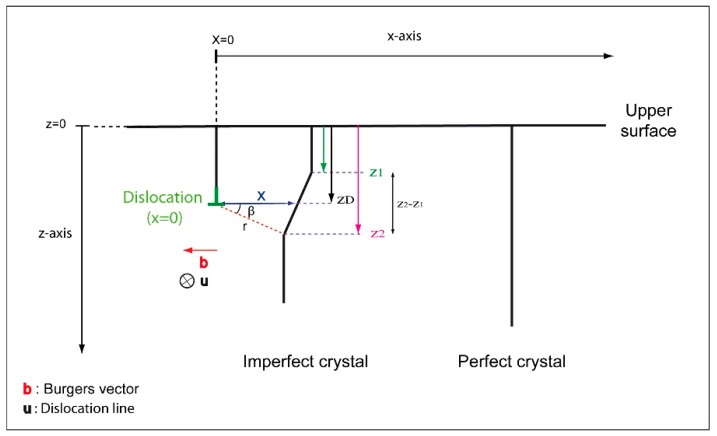
Schematic of an edge dislocation parallel to the surface and located at a depth z_D_. Deformed planes, perpendicular to the surface, are at a distance x away from the dislocation core.

Similar to the screw dislocation, an edge dislocation parallel to the surface and located at a depth z_D_ produces a local deformation of the crystalline planes nearby its core (see Figure 4). Such distortion is described by its displacement field, written in polar coordinate, as follows [15]:

(9) $${R\;}_{\mathrm{edge}}=\frac{b\;}{2\pi }\;\left[ß+\frac{\sin2ß}{2\left(1-\nu \right)}\right]+\;\frac{b\times u}{2\pi }[\frac{1-2\nu }{2\;\left(1-\nu \right)}\ln\left|r\right|+\;\frac{\cos2ß}{4\;\left(1-\nu \right)}]$$

ν is the Poisson’s ratio, u is the dislocation line direction, and r is the polar coordinates. Where ß and r are given by:

(10) $${ß=\tan}^{-1}(\frac{{z-z}_{D}}{x})\;\mathrm{and}\;r\;=\frac{x}{\cos ß}$$

From Equations (9) and (10), ${R\;}_{\mathrm{edge}}$ can be expressed as a function of the distance x away from the dislocation. The new deviation parameter s′ is then:

(11) $$s\prime \;=\;s\;+\;g{\cdot (\frac{{dR\;}_{\mathrm{edge}}}{\mathrm{dz}})}_{{z=z}_{D}}$$

The presence of an edge dislocation in the crystal can also be highlighted, analytically, by the insertion of Equation (11) in Equation (5). The calculated theoretical profiles are similar to that obtained for a screw dislocation. For the diffraction +g, at Bragg condition (s = 0), the modeled curves are characterized by a maximum and a minimum of I_BSE_. The edge dislocation generates a white/black contrast. However, for s > 0, the profile presents only a single peak consistent with experimental observations. The maximum intensity is situated at a position x ≈ −6 nm away from the dislocation core. Concerning the case of s < 0, the I_BSE_ profile show a hollow with a slight peak. All profiles are also reversed, following the inversion of the g sign regardless of the deviation parameter s.

### 2.3. Extinction Criteria

**Figure 5:**
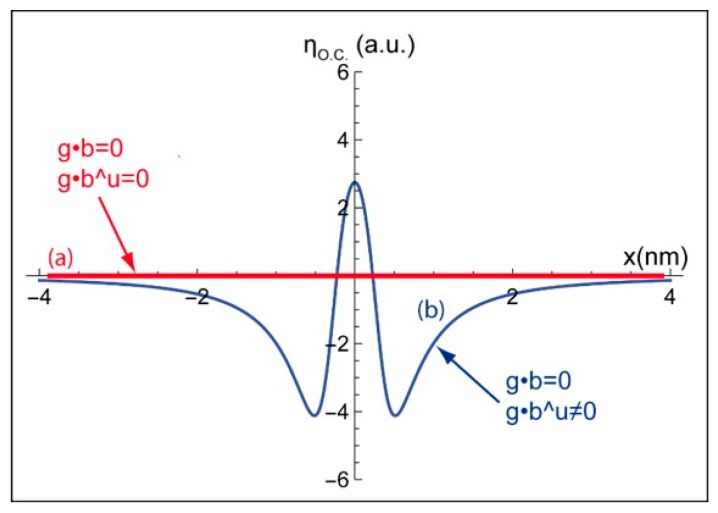
I_BSE_ profiles modeled for the extinction conditions: (a) g·b = 0, g·b×u = 0, and (b) g·b = 0, g·b×u ≠ 0.

Furthermore, for both screw and edge dislocations, considering the extinction criteria g·b = 0 and g·b×u = 0 in our equation leads to a null BSE yield ($\eta_{O.C.}=\;0\;a.u.$ in Figure 5a). Regarding the edge dislocation, the b×u term in Equation (9) becomes null when z = z_D_. Nevertheless, the position of the dislocation is located in the [z_1_, z_2_] range (see Figure 1), therefore the b×u term is not null. For g·b = 0 and g·b×u ≠ 0, in the [z_1_, z_2_] range, except z_D_, the calculated profile for an edge dislocation displays a low intensity peak $\eta_{O.C.}\approx \;2,7\;a.u$ (with respect to the background level) surrounded by two hollows. Such residual contrast (Figure 5b) is characteristic of an edge dislocation observed by TEM under these diffraction conditions [17].

### 2.4. Quantitative Comparisons with Experimental Profiles

**Figure 6:**
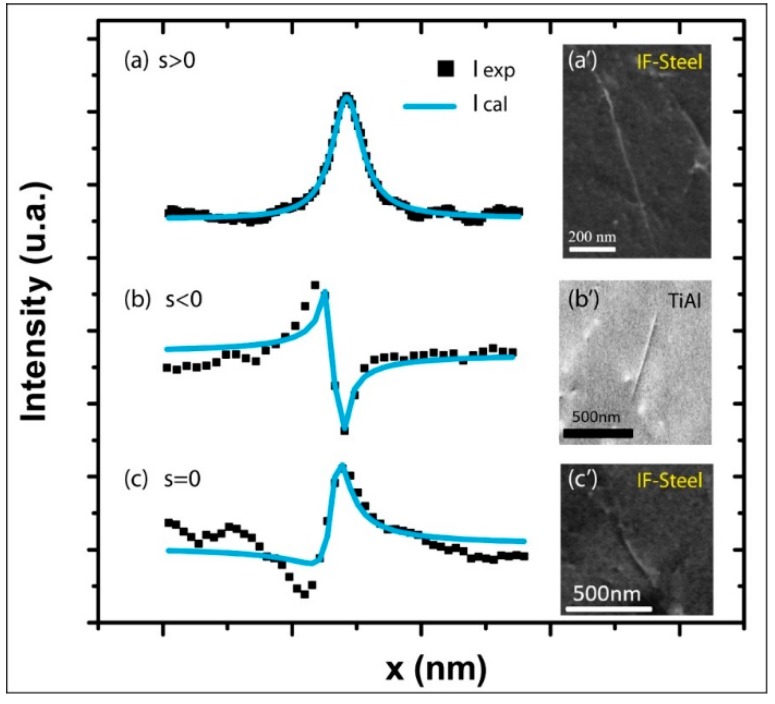
Fitted (blue line) and experimental (black squares) I_BSE_ profiles and their corresponding ECC micrographs obtained for (**a**,**a’**): **g** = (01-1) and s > 0, (**b**,**b’**): **g** = (020) and s < 0 and (**c**,**c’**): **g** = (2-1-1) and s = 0, respectively.

In this part, for each deviation parameter: s > 0, s < 0, and s = 0, an average profile is calculated from 50 experimental dislocation profiles and fitted by Equation (5). The results are illustrated by Figure 6a–c, respectively. 

As can be seen, the best fits are obtained for s>0 (the correlation coefficient $\chi^{2}\;$= 2) and s < 0 ($\chi^{2\;}$= 8.7). While for s = 0, the general features of the curve are well modeled, the correlation coefficient is higher: $\chi^{2}\;$ = 16.4. At Bragg condition, because of the strong interaction between the electron beam and the crystal atoms [18], dynamical effects are magnified, and the diffracted intensity is high enough to excite neighboring reflections. Then the successively and coherently produced beams interfere with each other. The “n” beam approach must thus be considered to better report the experimental results. Besides, in our calculations, multiple scattering was treated incoherently.

Nevertheless, the fitted profiles provide, among other parameters, reasonable orders of magnitude of the physical parameters $\xi_{g}$,${\;\xi }_{0}^{\prime }$, and $\xi_{g}^{\prime }$ for different deviation parameters and materials (interstitial Free (IF)-steel: Figure 5a,a’,c,c’ TiAl: Figure 6b,b’). Furthermore, the obtained parameters are in good agreement with the values reported in the literature [11]. Such as in the case of IF-steel: $\xi_{g}=9.4\;\pm \;1.3\;\mathrm{nm}$; $\xi_{0}^{\prime }=170.4\;\pm \;36.7\;\mathrm{nm}$; $\xi_{g}^{\prime }=177.7\;\pm \;38.3\;\mathrm{nm}$.

## 3. Conclusions

In this paper, an original theoretical model based on the Bloch wave approach of the dynamical diffraction theory was developed for modeling I_BSE_ around dislocations without resorting to numerical methods. An analytical formula of the BSE signal as a function of the different physical parameters governing the ECCI experiment has been proposed for the first time to our knowledge. The BSE contrast profiles, produced by screw and edge dislocations parallel to the sample surface, display the same appearance for the diffraction conditions. For a deviation parameter s = 0 (Bragg condition) and s < 0, the theoretical BSE curves show hollows and peaks of intensity corresponding to the black and white dislocation sides, respectively. The inversion of **g** leads to the profile inversion (hollow becomes peak and vice versa). For s > 0, the bright dislocation contrast is envisaged in the modeled profile by the intensity peak. The latter (dislocation image) does not coincide with the exact dislocation position (x = 0) and it is displaced to the opposite side when **g** is reversed. Moreover, our theoretical model confirms the use of the invisibility criteria in ECCI.

The good agreement between the theoretical and experimental results was also confirmed by adjusting the BSE intensity profiles. Hence, deduced values for the physical parameters $\xi_{g}$, the extinction distance, and $\xi_{0}^{\prime }$ and $\xi_{g}^{\prime }$, the absorption lengths, are consistent with the literature. Because the use of ECCI is becoming widespread for the defects characterization in bulk material in SEM, we provide a usable formula of the BSE intensity produced by dislocations. Furthermore, our approach can be extended to other defects. ECCI is now mature for exploring new horizons in materials science.
